# Role of Newly Introduced Teledentistry Service in the Management of Dental Emergencies During COVID-19 Pandemic in Qatar: A Cross-Sectional Analysis

**DOI:** 10.1089/tmj.2021.0584

**Published:** 2022-11-02

**Authors:** Shaymaa Abdulreda Ali, Abdul Mueen A. Al-Qahtani, Suhayla R. Al Banai, Fatima J. Albaker, Alanoud E. Almarri, Khalid Al-Haithami, Mohannad N. Khandakji, Walid El Ansari

**Affiliations:** ^1^ Unit of Orthodontics, Hamad Dental Center, Hamad Medical Corporation, Doha, Qatar.; ^2^ Unit of Prosthodontics, Hamad Dental Center, Hamad Medical Corporation, Doha, Qatar.; ^3^ Al Wakra Dental Hospital, Hamad Medical Corporation, Doha, Qatar.; ^4^ Unit of Endodontics, Hamad Dental Center, Hamad Medical Corporation, Doha, Qatar.; ^5^ Department of Surgery, Hamad General Hospital, Hamad Medical Corporation, Doha, Qatar.; ^6^ College of Medicine, Qatar University, Doha, Qatar.; ^7^ Weill Cornell Medicine—Qatar, Doha, Qatar.; ^8^ School of Health and Education, University of Skovde, Skovde, Sweden.

**Keywords:** dental emergency, access to care, telemedicine, teledentistry, COVID-19, teletriage

## Abstract

**Introduction:**

The lockdown imposed by the COVID-19 pandemic rendered teledentistry (TD) necessary to maintain the continuity of oral health services and avoid missing emergency dental conditions, while minimizing face-to-face visits. Our objective was to evaluate the ability of a newly introduced triage-based TD service to deliver its goals, by evaluating its processes and outcomes and assessing the demand for TD.

**Methods:**

This cross-sectional report assessed the triage processes and outcomes (triage category, referral to emergency/dental facility undertaken, remote medications prescribed, and procedures performed at the point of referral); and evaluated the demand for the newly introduced TD service during 5 months of the first wave of the pandemic.

**Results:**

Of 850 calls, about 70.6% of the samples were managed remotely; 29.4% were categorized as emergency/urgent and referred to the emergency/dental facility. Compared with other complaints, orofacial dental pain was the most common reason for the calls (41.6%,* p *< 0.0001). About 14.71% of callers received prescription for medications remotely. The most demanded disciplines were general dentistry, orthodontics, and oral surgery, respectively (*p *< 0.0001). Of those referred to a dental facility, 31.84% required no clinical intervention, 28.7% received orthodontic appliance repair, and 14.3% and 11.2% had urgent dental extractions or root canal treatments. Demand on the service fluctuated through various distinct stages of the lockdown.

**Conclusions:**

There has been continuous demand for the newly introduced TD service throughout the period of the current report despite the fluctuations, with most complaints managed remotely. TD was effective and suitable for triage, service delivery, and care during the pandemic.

## Introduction

As COVID-19 spread across the globe, social distancing and restrictions were imposed to limit the spread of the disease. The state of Qatar responded by a nationwide lockdown since March 9, 2020. This measure limited dental services to emergency and urgent care and led to the postponement of elective services due to the risk of virus transmission associated with the aerosols generated during dental procedures.^1^ Hence, instead of the traditional face-to-face care, teledentistry (TD) was initiated for the early management of acute dental conditions.

TD, the remote provision of dental care without physical contact, is not a specific service, but rather, a set of technologies and tactics to enhance care delivery.^2,3^ TD, including telephone triage, allows practitioners to interview callers, assess urgency, and sort patients by priority and level of care required.^4,5^ Globally, reports of the pandemic's impact on the delivery of urgent dental care recommend that telephone triage be mandatory in the assessment of the need for emergency treatment.^6–8^ In response, the Hamad Dental Center (HDC), the only public tertiary dental care provider in Qatar, initiated a dedicated TD hotline to ensure the continuity of safe effective care.^9^

While TD consultations can identify oral diseases and guide referrals,^10^ the literature on TD and telephone triage during the pandemic reveals knowledge gaps. These have to do with the limited scope, short duration, restricted patient populations, and limited geographical coverage. In terms of scope, no previous TD research appraised the details of the call, patient, condition, or the triage process and its outcomes. Previous studies examined selective conditions, for example, dental-facial trauma,^11^ or pain, swelling, and trauma,^12^ with less attention to, for example, loose/broken dental restorations, orthodontic appliances, oral ulcers, or bleeding that might lead to serious consequences.

Likewise, studies were of short duration (e.g., 1 month^12^), not reflecting the changes in demand on TD by stages of the pandemic. In addition, the majority of reports focused on pediatric populations,^11,13^ despite that TD is appropriate for the elderly and those with chronic conditions, known to have increased risk of infection.^14,15^ Geographically, most pandemic-related publications on TD and telephone triage remain from the United States, Europe, or Asia,^11–14,16–18^ with very few reports from the Middle East and North Africa region. This is despite that, for example, in Saudi Arabia, 35.2% of the patients sought dentistry teleconsultation as the first step during the pandemic.^19^

Therefore, the current study assessed the broader scope (process and outcomes) of a TD service provided to a representative sample (adult and pediatric populations) in Qatar during a long duration (5 months of the pandemic's first wave, March 29 to August 31, 2020).

The report appraised selected characteristics of the following: (1) call (frequency, time, and duration); and (2) caller (demographics, medical history, and relationship of caller with patient). For all HDC hotline calls, the specific objectives were to appraise the ability of the newly introduced TD triage-based management approach to deliver the service goals by (1) evaluating the triage processes (triage category, referral to emergency/dental facility undertaken, and categorization by discipline required) and outcomes (medications prescribed and procedure performed at the point of referral); and (2) evaluating the demand for the newly introduced TD service.

The findings of the current study bridge the knowledge gaps and offer evidence-based guidance for policy makers and providers to avoid complications of emergency/urgent oral/dental conditions.^20^ The present appraisal of our TD experience will inform the future commissioning and development of such services in Qatar and worldwide.

## Methods

### ETHICS, DESIGN, AND PARTICIPANTS

This service evaluation project was granted permission to proceed from our hospital institutional review board. It comprised a retrospective analysis of data routinely collected for clinical audit and as an integral part of service evaluation purposes. We analyzed call, patient, and triage data of all calls during the first wave of the COVID-19 lockdown (*N* = 1,239 during 5 months); and excluded callers with incomplete records (*N* = 389), leaving 850 calls included in this service evaluation.

### SETTING AND PROCEDURES

A team of qualified dentists formally set up the HDC hotline to maintain the continuity of services and avoid missing life-threatening or emergency dental conditions. The team's senior members prepared algorithms to categorize the triage levels. Protocols were also prepared to assist the hotline dentists in the management of self-reported complaints, for example, pain, swelling, bleeding, trauma, and oral-mucosal ulceration. The algorithms and protocols were based on international recommendations,^2,21,22^ and were aimed to assist the tele-dentist to triage the call, arrive at provisional diagnosis, and provide appropriate remote care or referral.

In addition, we retrieved data collected by the dentists as part of the service using the teledentistry form (TDF) that included the information on the following: (1) call (frequency, time, and duration); (2) patient (demographics, medical/allergy history, relationship of caller with patient, chief complaint, and severity of pain on a scale from 0 to 10); and (3) triage (specialty required, management, referral to emergency/dental facility undertaken, and medications prescribed), in line with others.^23–25^ The TDF was available to each dentist, posted near the telephone set; the dentist completed it, and used it with the algorithms to make decisions. When required, medications were home delivered to patients using courier service. The 11 hotline dentists had received training on all the above to ensure consistency of the service, and had a dedicated workspace, where caller privacy was observed.

### STATISTICAL ANALYSIS

Descriptive and inferential statistics characterized the sample. Categorical variables are reported as frequencies and percentages and compared using the chi-squared test. *p-*Value <0.05 (two tailed) was considered statistically significant.

## Results

### SELECTED CHARACTERISTICS OF THE SAMPLE AND THE HOTLINE CALLS

The number of female callers was slightly more than male callers (*Table 1*). Most callers were aged 18–49 years, almost half were Qatari nationals, and 30% were from other Arab nations. Slightly more than half the callers reported no previous medical history, 12.94% and 8.35% had allergy and diabetes, respectively, and few had history of hypertension, and cardiovascular or endocrine conditions (3.07%–5.78%). *Table 1* also shows that one-third of the sample required a family member/caregiver to mediate the call. Most patients called during the morning (7 am–2 pm). Mean duration of the call was 5.36 ± 3.37 min (range 1–38 min). About 10% of the callers made repeated calls, and among the repeated calls, 15 patients reported a different oral/dental complaint not related to the first complaint.

**Table 1.** Selected Characteristics of the Sample and the Hotline Calls

CHARACTERISTIC	*N* (%)
Sex	
Female	456 (53.65)
Male	394 (46.35)
Age, years	
<18	165 (19.41)
18–29	183 (21.53)
30–39	201 (23.65)
40–49	129 (15.18)
50–59	95 (11.18)
60–69	53 (6.24)
≥70	24 (2.82)
Nationality	
Qatari	419 (49.29)
Arab	255 (30)
Asian	144 (16.94)
Other	32 (3.76)
Medical history	
Allergy	110 (12.94)
Diabetes	71 (8.35)
Hypertension	49 (5.76)
Asthma	39 (4.59)
Cardiovascular	33 (3.88)
Endocrine	26 (3.06)
Other^a^	68 (8.0)
None	454 (53.41)
Call conducted by patient	
Yes	609 (71.69)
No	241 (28.31)
Time of call	
7 am–2 pm	543 (63.9)
2 pm–10 pm	307 (36.1)
Duration of call, min	
Mean ± standard deviation	5.36 ± 3.37
Range	1–38 min
Repeated call	
Yes	86 (10.12)
No	764 (89.88)

^a^ Includes pulmonary, dermal, mental, gastrointestinal, renal, neurological, and musculoskeletal conditions, cancer; 10-year age brackets used in line with the WHO.

### TRIAGE PROCESSES AND OUTCOMES

**Fig. 1.**
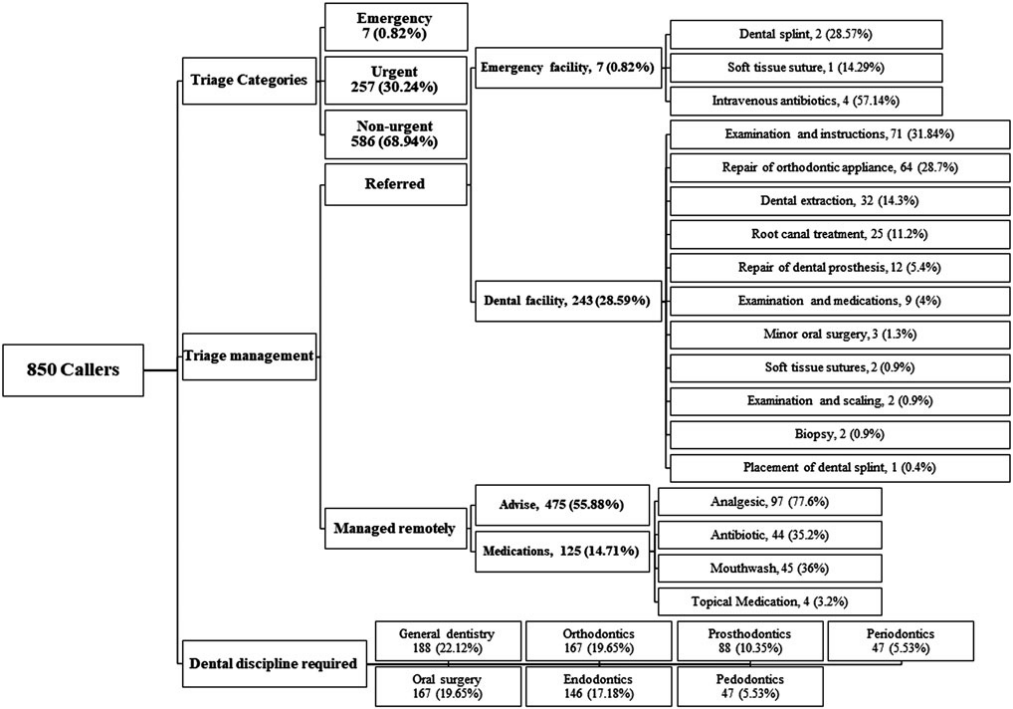
Summary of triage processes and outcomes.

*Figure 1* summarizes the triage processes and outcomes. *Table 2* illustrates that almost a third of the calls were categorized as emergency/urgent, and the remaining two-thirds were triaged as nonurgent, where 70.59% of calls were manageable remotely by advice, care and oral hygiene instructions, or medications. In terms of management, almost a third of the complaints were not manageable by TD and were referred to a hospital emergency or dental facility to receive emergency/urgent clinical procedure. Most of these procedures required a general dentist, followed by orthodontics, oral-maxillofacial, and endodontics.

**Table 2.** Triage Processes

PROCESS	*N* (%)
Triage	*N* = 850
Categories	
Emergency	7 (0.82)
Urgent	257 (30.24)
Nonurgent	586 (68.94)
Management	
Instructions	475 (55.88)
Medications	125 (14.71)
Referred to	250
Dental facility	243 (28.59)
Hospital emergency facility	7 (0.82)
Categorization by discipline required	*N* = 850
General dentistry	188 (22.12)
Orthodontics	167 (19.65)
Oral surgery	167 (19.65)
Endodontics	146 (17.18)
Prosthodontics	88 (10.35)
Pedodontics	47 (5.53)
Periodontics	47 (5.53)

Of the patients referred for face-to-face treatment, 20 did not show up at the point of referral, 7 attended a hospital emergency facility, and 223 patients attended a dental facility (*Table 3*). Most of those who attended at hospital (4 out of 7 patients) received intravenous antibiotics to control facial cellulitis of dental origin. *Table 3* illustrates that after a clinical examination, almost one-third of the 223 patients who visited the dental facility required no clinical intervention, and a minority (4%) required medications. Of those who visited the dental facility, more than a quarter received an orthodontic appliance repair procedure, while urgent dental extractions and root canal treatments were less frequent. All patients who were referred at the triage stage subsequently received follow-up appointments from their point of referral.

**Table 3.** Triage Outcome (Procedure Received at Point of Referral)

PROCEDURE	*N* (%)
All procedures	230
At hospital emergency facility	7 (100)
Dental splint	2 (28.57)
Soft tissue suture	1 (14.29)
Intravenous antibiotics	4 (57.14)
At dental facility	223 (100)
Examination, reassurance, and instructions	71 (31.84)
Repair of orthodontic appliance	64 (28.7)
Dental extraction	32 (14.3)
Root canal treatment	25 (11.2)
Repair of dental prosthesis	12 (5.4)
Examination and medications	9 (4)
Minor oral surgery	3 (1.3)
Soft tissue sutures	2 (0.9)
Examination and scaling	2 (0.9)
Biopsy	2 (0.9)
Placement of dental splint	1 (0.4)

A total of 14.71% out of the samples were prescribed medications remotely. Of these, the most frequent prescribed medication was analgesics (77.6%) (*Table 4*). Some callers received a combination of medications (58 patients, data not presented). Based on the chief complaint, most prescriptions were for those who reported dental pain and swelling. Endodontic-related complaints received 37% of prescriptions, followed by oral surgery and general dentistry concerns (19.20% and 17.60%, respectively). Most analgesics were prescribed to manage dental/orofacial pain, while antibiotics were prescribed to manage either oral/facial swelling and/or associated dental/orofacial pain (40.91%).

**Table 4.** Triage Outcome (Medications Prescribed Remotely)

VARIABLE	TOTAL	MEDICATION, *N* (%)^a^			
		ANALGESIC	ANTIBIOTIC	MOUTHWASH	TOPICAL
All patients who received medications remotely	125 (100)	97 (77.6)	44 (35.2)	45 (36.0)	4 (3.2)
Patients who received medications by chief complaint					
Pain	72 (57.6)	64 (65.98)	18 (40.91)	18 (40.0)	1 (25.0)
Swelling	36 (28.8)	23 (23.71)	26 (59.09)	16 (35.56)	0 (0)
Problem related to dental restoration/prosthesis	6 (4.8)	4 (4.12)	0 (0)	4 (8.89)	0 (0)
Bleeding	6 (4.8)	3 (3.09)	0 (0)	5 (11.11)	0 (0)
Ulcer	3 (2.4)	1 (1.03)	0 (0)	2 (4.44)	3 (75.0)
Broken/loose/or decayed tooth	2 (1.6)	2 (2.06)	0 (0)	0 (0)	0 (0)
Problem related to orthodontic appliance	0 (0)	0 (0)	0 (0)	0 (0)	0 (0)
Trauma	0 (0)	0 (0)	0 (0)	0 (0)	0 (0)
Appointment enquiry	0 (0)	0 (0)	0 (0)	0 (0)	0 (0)
Patients who received medications by specialty required					
Endodontic	47 (37.6)	43 (44.33)	24 (54.55)	16 (35.56)	0 (0)
Oral surgery	24 (19.2)	20 (20.62)	6 (13.64)	9 (20.0)	3 (75.0)
General dental	22 (17.6)	17 (17.53)	6 (13.64)	4 (8.89)	0 (0)
Periodontic	14 (11.2)	5 (5.15)	4 (9.09)	10 (22.22)	1 (25.0)
Pedodontics	9 (7.2)	7 (7.22)	4 (9.09)	1 (2.22)	0 (0)
Orthodontic	5 (4.0)	3 (3.09)	0 (0)	3 (6.67)	0 (0)
Prosthodontic	4 (3.2)	2 (2.06)	0 (0)	2 (4.44)	0 (0)

^a^ For the first row, percentages between brackets are based on the total number of patients who were prescribed medications remotely (*n* = 125); percentages do not add up to 100% as some patients were prescribed >1 type of medication. The remaining percentages are based on the number of patients in the header of each respective column.

Medications prescribed by chief complaint and specialty required.

Across the whole sample (*Table 5*), for most calls, the chief complaint was pain, followed by problems related to dental restorations or oral/facial swelling. One-third of callers with pain were categorized as emergency/urgent, and about a quarter of those with pain were referred to an emergency/dental facility for treatment. While the least frequent reported conditions were oral ulcers and dental/oral soft tissue trauma (2.24% and 1.76%, respectively), about half of these (42.11% and 46.67%, respectively) were categorized as emergency/urgent and referred face-to-face management.

**Table 5.** Triage Categories and Referrals by Chief Complaint and Specialty Required

VARIABLE	TOTAL^a^	TRIAGE OUTPUT					
		CATEGORIZED AS EMERGENCY/URGENT			REFER TO EMERGENCY/DENTAL FACILITY		
		NO	YES	*p*	NO	YES	*p*
All callers	850 (100)	583 (68.59)	267 (31.41)	<0.0001	600 (70.6)	250 (29.4)	<0.0001
By chief complaint				<0.0001			<0.0001
Pain	349 (41.6)	235 (67.34)	114 (32.66)		258 (73.9)	91 (26.10)	
Dental restoration^b^	146 (17.18)	88 (60.27)	58 (39.73)		85 (58.2)	61 (41.80)	
Swelling	109 (12.82)	69 (63.30)	40 (36.7)		69 (63.3)	40 (36.70)	
Orthodontic appliance	85 (10.00)	67 (78.82)	18 (21.18)		65 (76.5)	20 (23.50)	
Appointment enquiry	58 (6.82)	54 (93.10)	4 (6.90)		53 (91.4)	5 (8.6)	
BLD tooth	44 (5.18)	33 (75.00)	11 (25.00)		33 (75.0)	11 (25.0)	
Bleeding	25 (2.94)	18 (72.00)	7 (28.00)		18 (72.0)	7 (28.0)	
Ulcer	19 (2.24)	11 (57.89)	8 (42.11)		11 (57.9)	8 (42.1)	
Trauma	15 (1.76)	8 (53.33)	7 (46.67)		8 (53.3)	7 (46.7)	
By specialty required				<0.0001			<0.0001
GDP	188 (22.12)	152 (80.85)	36 (19.15)		157 (83.51)	31 (16.49)	
Oral surgery	167 (19.65)	79 (47.30)	88 (52.7)		90 (53.89)	77 (46.11)	
Orthodontics	167 (19.65)	111 (66.47)	56 (33.53)		105 (62.87)	62 (37.13)	
Endodontics	146 (17.18)	99 (67.81)	47 (32.19)		108 (73.97)	38 (26.03)	
Prosthodontics	88 (10.35)	67 (76.14)	21 (23.86)		65 (73.86)	23 (26.14)	
Pedodontics	47 (5.53)	37 (78.72)	10 (21.28)		37 (78.72)	10 (21.28)	
Periodontics	47 (5.53)	38 (80.85)	9 (19.15)		38 (80.85)	9 (19.15)	

^a^ Percentages in this column are column percentages.

^b^ Either filling or prosthodontic restoration.

BLD, broken/loose/decayed; GDP, general dental practitioner.

Cells represent frequency and row percentages except where indicated.

As for the specialty required, more than 50% of the complaints requiring oral surgery were categorized as emergency/urgent and were transferred to an emergency/dental facility for chairside procedures. Around one-third of callers who required endodontics and orthodontic care were considered emergency/urgent and were referred for face-to-face management.

### DEMAND FOR TD

**Fig. 2.**
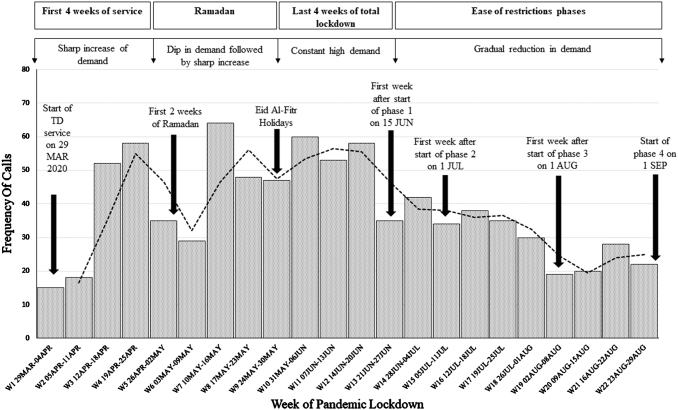
Frequency of calls by week of pandemic.

The frequency of calls by week during the first wave of the pandemic reflects the demand for the service (*Fig. 2*). Demand steadily increased during the first 2 weeks, followed by a steep rise by week 3, then a sudden dip during weeks 5–6 coinciding with the beginning of fasting of the Holy Month of Ramadan. As Ramadan progressed, demand escalated again starting from week 7. In week 9, there was another brief decrease in demand coinciding with the holidays of Holy Eid El-Fitr that marks the end of Ramadan. This was followed by an increase in demand again until the lockdown measures started to ease at the beginning of week 13 (June 15, 2020), where it slowly decreased reflecting the resumption of work at almost all private dental practices. Demand then leveled off, maintaining a level of about 18–20 calls per week.

## Discussion

Our TD service was initiated to identify and address emergency/urgent oral and/or dental complaints and avoid missing life-threatening conditions. TD was not utilized at HDC before the pandemic and with the start of the service on March 29, 2020, demand for TD steeply increased, and then fluctuated at various distinct stages of the lockdown. There is scarce literature published on the topic with which we could directly compare our observations with.

In terms of limiting face-to-face visits to emergency/urgent conditions, the TD service managed two-thirds of the calls remotely, significantly reducing the number of patients who would have otherwise physically visited HDC. Such decrease concurs with the high reduction (49.3%) in patient flow observed elsewhere.^12^ These cutbacks are critical as they minimize the risk of COVID-19 transmission. We observed that only 29.4% of the callers were referred to emergency/dental facility, less than referrals reported from Belgium, the United Kingdom, and the United States, where telephone triage referred 50.7%–61% of calls to an urgent care center during the pandemic.^12,16,17^ Such a higher referral rate observed by others might be due to the shorter durations of observation that do not consider changes in demand, lower number of calls, higher number of service providers, as well as differences in triage algorithms due to the breadth of the definitions of dental emergency, urgent, and nonurgent dental conditions.^20^

We support recent proposals for the need of evidence premised on different demographic profiles and countries to better define dental interventions that fall in the basic, urgent, or emergency categories of essential oral health care.^20^

As for complaints and associated procedures, 41.6% of the callers reported dental pain, close to the 45.9%–54% identified by others.^12,16^ Likewise, our callers reported a trauma rate of 1.76%, akin to the 2.3%–2.7% described elsewhere.^12,16^ Nevertheless, we observed a 12.82% rate of swelling, considerably higher than other studies (1.7%).^16^ While others did not examine problems related to broken/dislodged orthodontic appliance or dental restorations,^12,16^ 20% of the HDC calls required orthodontic expertise and were referred to a dental facility, where the most common treatment was orthodontic appliance repair (28.7%).

Other reports similarly found that orthodontic emergencies increased at walk-in emergency dental facilities during the pandemic compared with the prepandemic levels.^26^ Although such emergencies may not be life threatening, they challenge daily activities (speech, mastication, and sleep), quality of life, and could lead to significant consequences if unattended.

Notwithstanding, while other studies found dental extractions to be the most provided treatment at dental facilities during the pandemic (63%),^17^ our corresponding rate was 14.3%. Carter highlighted that their high dental extraction rate was due to their adherence to avoid aerosol generating procedures where possible, and to a high prevalence of open dental pulps and oral sepsis in their population, comprising some of lowest socioeconomic groups in England.^17^ Our center similarly limited aerosol generating procedures evident by the low levels (11.2%) of emergency root canal treatments that were only conducted when the tooth was restorable and strategically important.

In terms of prescription of medications for calls that were remotely managed, medications were utilized for only 14.71% of these calls. Similarly, antibiotics were prescribed for only 5.15% of the total sample, which is significantly lower than levels (47%) reported by others,^27^ suggesting that HDC tele-dentists reserved the use of medications for the more painful conditions.

In terms of demand for the TD service, the sudden initial escalation of demand for TD we observed was also noted elsewhere following the publication that dental services were to be provided by telephone only.^21^ Similarly, the fluctuation in demand we noticed is consistent with other reports,^28^ and explained by changes in patient health-seeking behavior during pandemics.^29,30^ In addition, our TD service maintained a relatively high demand during and after phase 3 of restriction ease, suggesting that it was valued in the provision of oral health services during and after the lockdown. Such remote management and sustained demand highlight the potential of TD after the pandemic subsides.

Regarding the characteristics of the calls (time and duration), such information is valuable when planning TD workforce and duty roosters. The patient characteristics reveal that a third of our sample required a family member, guardian, or caregiver to mediate the call, probably because some callers were non-Arabs (20.7%), <18 years old (19.8%), or elderly (9%). In addition, TD traditionally comprised a trained dental professional at one end interacting with a dental assistant/hygienist and the patient at, for example, a remote clinic or health post.^3^

During the current pandemic, TD changed to be directly between the dental professional and patient, hence probably necessitating the mediation of a family member, guardian, or caregiver in some instances. A point is that while teleassessment within the home environment is feasible for many medical conditions (e.g., children with autism^31,32^), TD could represent additional challenges. From the provider side, training for dentists is required to enhance their ability to provide precise information to patients/caregivers to perform delicate tasks, for example, compression to stop bleeding or undertaking oral hygiene measures. Likewise, patients/caregivers need to have the ability and willingness to administer the required tasks and provide real-time feedback to the tele-dentist.

The current report has some limitations. Missing data are common in retrospective interrogations of data sets routinely collected as part of the service. In addition, at HDC, not all the potential of TD was initially mobilized. For instance, most patients called in via telephone, and so, audio-dentistry comprised a major part of the service, as videos were not conducted and only a few patients sent images. Audio-dentistry during the early days of the pandemic is not uncommon,^16^ because major reorganization of dental care services is a challenge that requires time.^12,33^ In addition, not all callers are tech literate or comfortable with a new approach of interaction with the service^16^ or with sending personal photographs.

Currently, HDC has the setup, facilities, software, videoconferencing capabilities, and other resources required for full-blown TD. Despite this, the current report has many strengths. It assessed a broad scope of a new service provided to a large sample (adult and pediatric populations) during a long duration (5 months), with an appraisal of the epidemiology of a wide range of variables, including call, patient, and triage characteristics. Furthermore, the callers captured in this report are representative of the demographically and ethnically diverse population in Qatar as HDC is the sole public tertiary dental care provider. Hence, the current report comprised nationals and a large diverse multinational sample with different genetic backgrounds, which makes the generalization of the findings possible.

## Conclusions

The primary goal of the new TD service at HDC was to maintain the continuity of oral health services for emergency dental conditions only, in order to minimize face-to-face visits during the pandemic. Although TD was new at HDC, the service setup and operation went smoothly, and the tele-dentists were able to deliver the service objectives. Telephone triage proven to be useful in the assessment of the need for emergency treatment and most complaints were managed remotely.

During the critical times of the first wave of the COVID-19 pandemic lockdown in Qatar, there was a persistent demand for the HDC TD service throughout the report period. It should be straightforward to set up and utilize such service effectively in any dental setting, however, for future similar circumstances, it is important to plan the service, considering that the policies and guidelines allowed for consistent service delivery. Moreover, orofacial dental pain was the most common reason for the calls; and that the most demanded disciplines were general dentistry, followed by orthodontics and oral surgery, with the most demanded procedures being repairs of fixed orthodontic appliances, urgent extractions, and urgent root canal treatments.
